# Low concentrations of fine particle air pollution and mortality in the Canadian Community Health Survey cohort

**DOI:** 10.1186/s12940-019-0518-y

**Published:** 2019-10-10

**Authors:** Tanya Christidis, Anders C. Erickson, Amanda J. Pappin, Daniel L. Crouse, Lauren L. Pinault, Scott A. Weichenthal, Jeffrey R. Brook, Aaron van Donkelaar, Perry Hystad, Randall V. Martin, Michael Tjepkema, Richard T. Burnett, Michael Brauer

**Affiliations:** 1 Health Analysis Division, Statistics Canada, 100 Tunney’s Pasture Driveway, Ottawa, Ontario K1A 0T6 Canada; 2 School of Population and Public Health, The University of British Columbia, 2206 East Mall, Vancouver, British Columbia V6T 1Z3 Canada; 3 Department of Sociology, University of New Brunswick, PO Box 4400, Fredericton, New Brunswick E3B 5A3 Canada; 4 Department of Epidemiology, Biostatistics & Occupational Health, McGill University, 1110 Pine Ave West, Montreal, Quebec H3A 1A3 Canada; 5 Air Health Science Division, Health Canada, 269 Laurier Avenue West, Ottawa, Ontario K1A 0K0 Canada; 6 Dalla Lana School of Public Health, University of Toronto, 155 College Street, Toronto, Ontario M5T 1P8 Canada; 7 Department of Physics and Atmospheric Science, Dalhousie University, 6310 Coburg Road, PO Box 15000, Halifax, NS B3H 4R2 Canada; 8 College of Public Health and Human Sciences, Oregon State University, 2520 SW Campus Way, Corvallis, Oregon 97331 USA; 9 Harvard-Smithsonian Center for Astrophysics, 60 Garden St, Cambridge, MA 02138 USA; 10 Population Studies Division, Health Canada, 50 Columbine Driveway, Ottawa, Ontario K1A 0K9 Canada; 11 Department of Energy, Environmental & Chemical Engineering, Washington University in St. Louis, St. Louis, Missouri 63130 USA; 12 Department of Chemical Engineering and Applied Chemistry, University of Toronto, 223 College St., Toronto, ON M5T 1R4 Canada; 13 Safe Environments Directorate, Health Canada, 269 Laurier Avenue West, Ottawa, Ontario K1A 0K9 Canada

**Keywords:** PM_2.5_, Air pollution, Canada, Cohort study, Fine particulate matter, Mortality, Fine particle air pollution

## Abstract

**Background:**

Approximately 2.9 million deaths are attributed to ambient fine particle air pollution around the world each year (PM_2.5_). In general, cohort studies of mortality and outdoor PM_2.5_ concentrations have limited information on individuals exposed to low levels of PM_2.5_ as well as covariates such as smoking behaviours, alcohol consumption, and diet which may confound relationships with mortality. This study provides an updated and extended analysis of the Canadian Community Health Survey-Mortality cohort: a population-based cohort with detailed PM_2.5_ exposure data and information on a number of important individual-level behavioural risk factors. We also used this rich dataset to provide insight into the shape of the concentration-response curve for mortality at low levels of PM_2.5_.

**Methods:**

Respondents to the Canadian Community Health Survey from 2000 to 2012 were linked by postal code history from 1981 to 2016 to high resolution PM_2.5_ exposure estimates, and mortality incidence to 2016. Cox proportional hazard models were used to estimate the relationship between non-accidental mortality and ambient PM_2.5_ concentrations (measured as a three-year average with a one-year lag) adjusted for socio-economic, behavioural, and time-varying contextual covariates.

**Results:**

In total, 50,700 deaths from non-accidental causes occurred in the cohort over the follow-up period. Annual average ambient PM_2.5_ concentrations were low (i.e. 5.9 μg/m^3^, s.d. 2.0) and each 10 μg/m^3^ increase in exposure was associated with an increase in non-accidental mortality (HR = 1.11; 95% CI 1.04–1.18). Adjustment for behavioural covariates did not materially change this relationship. We estimated a supra-linear concentration-response curve extending to concentrations below 2 μg/m^3^ using a shape constrained health impact function. Mortality risks associated with exposure to PM_2.5_ were increased for males, those under age 65, and non-immigrants. Hazard ratios for PM_2.5_ and mortality were attenuated when gaseous pollutants were included in models.

**Conclusions:**

Outdoor PM_2.5_ concentrations were associated with non-accidental mortality and adjusting for individual-level behavioural covariates did not materially change this relationship. The concentration-response curve was supra-linear with increased mortality risks extending to low outdoor PM_2.5_ concentrations.

## Background

Exposure to ambient fine particle air pollution (PM_2.5_) is responsible for an estimated 2.9 million deaths annually and 83 million disability-adjusted life years lost [1], with several large epidemiological cohort studies linking long-term exposure to PM_2.5_ to all-cause and cause-specific mortality [2–4]. Even in settings with relatively low concentrations of air pollution, such as Canada, the relationships persist [5, 6]. Despite these findings, there remain two key areas of potential bias and uncertainty that past work has been unable to address simultaneously. The first is the inability to directly adjust for individual-level behavioural risk factors associated with chronic disease mortality, such as smoking, diet, and exercise, or health measures such as body mass index; various indirect methods for adjustment have been applied elsewhere [7, 8]. The second regards the shape of the concentration-response curve for PM_2.5_ and mortality. This issue has become increasingly pertinent as clean air regulations have succeeded in reducing PM_2.5_ concentrations across North America and elsewhere, and thus a better understanding of the shape of the PM_2.5_-mortality associations at low concentrations are required for cost-benefit assessments of future reduction efforts.

The purpose of this study was to provide an updated and extended analysis of the Canadian Community Health Survey-Mortality cohort [9] including [1]: additional years of follow-up to 2016 [2]; improvements in the resolution of PM_2.5_ exposure (approximately 1km^2^ grid) [3]; annual residential history from 1981 to 2016 for all cohort members from a linkage to postal code records [4]; time-varying contextual covariates [5]; inclusion of immigrants to Canada, and [6] an improved linkage between survey respondents and death records. We examine the shape of the concentration-response curves using a Shape Constrained Health Impact Function (SCHIF) [10] and perform sensitivity analyses.

## Methods

### CCHS-mortality cohort

**Fig. 1:**
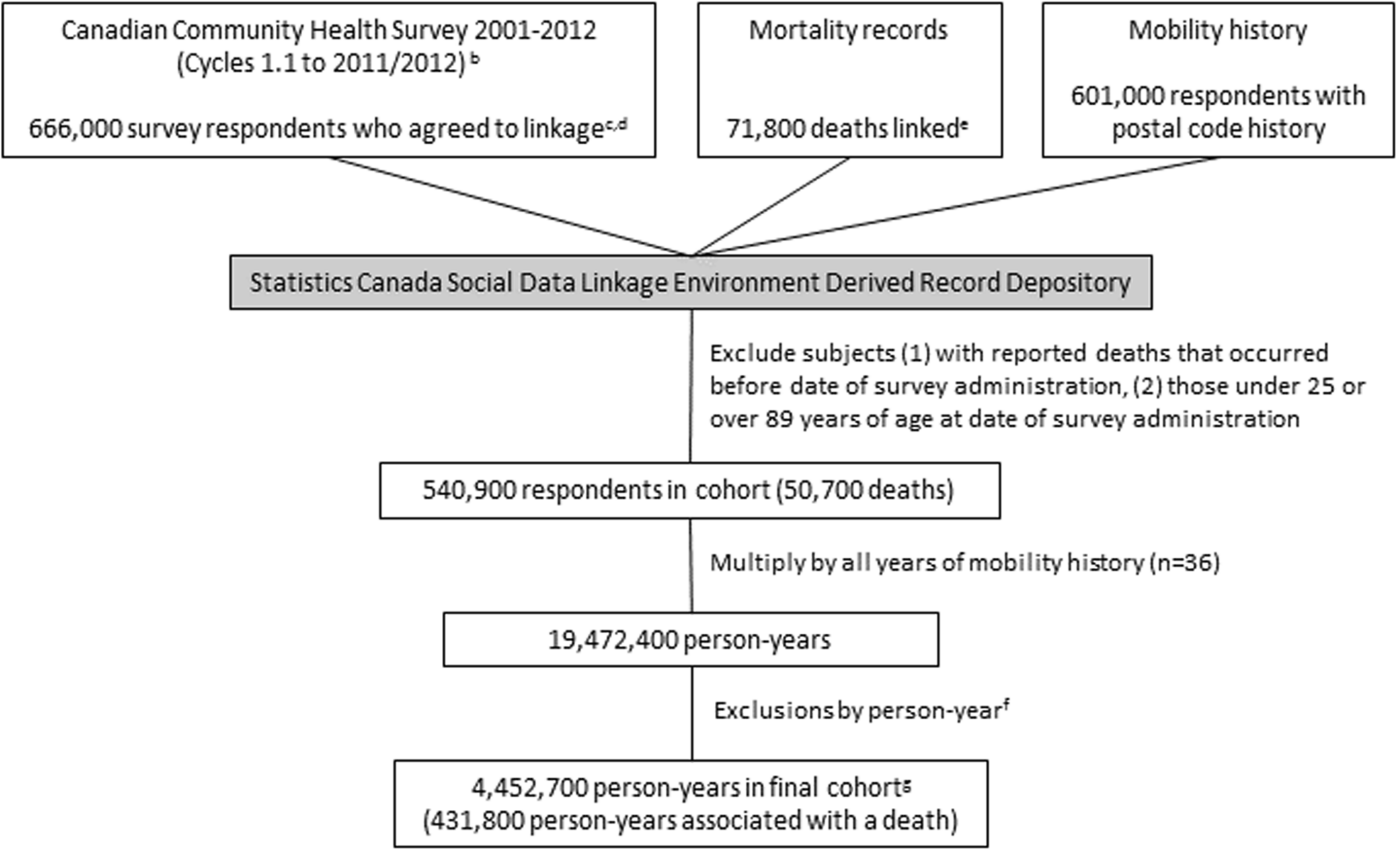
Flowchart of CCHS-Mortality cohort creation from linkage of survey to mortality and mobility history to person-year based analytical file^a^. ^a^numbers rounded to the nearest 100 for confidentiality. ^b^response rates: 2000/2001 (Cycle 1.1) 84.7%, 2003 (Cycle 2.1) 80.7%, 2005 (Cycle 3.1) 78.9%, 2007/2008 (Cycle 4.1) 76.4%, 2009/2010 72.3%, 2011/2012 68.4%. ^c^respondents who agreed to data linkage: 2000/2001 (Cycle 1.1) *n* = 117,800, 2003 (Cycle 2.1) *n* = 112,900, 2005 (Cycle 3.1) *n* = 113,900, 2007/2008 (Cycle 4.1) n = 112,700, 2009/2010 *n* = 104,700, 2011/2012 n = 104,100. ^d^linkage rate of respondents who agreed to linkage to the SDLE DRD: 95.2%. ^e^linkage rate of relevant deaths to the SDLE DRD: 99.8%. ^f^see methods for list of exclusion criteria, totals will exceed number of deleted person-years given that more than one exclusion criteria may apply to a single person-year; immigrated to Canada less than 10 years before survey date *n* = 541,600, age during follow-up period exceeds 89 years *n* = 161,000, no postal code n = 5,009,900, could not be linked to air pollution values n = 5,711,600, could not be linked to Can-MARG values *n* = 7,668,000, could not be linked to Census Metropolitan Area/Census Agglomeration size *n* = 4,800,600, could not be linked to airshed *n* = 3500, 3-year moving average being informed by only 1 year of exposure *n* = 4,321,500, year after subject death *n* = 343,600, year before survey interview date *n* = 13,570,300. ^g^from 452,700 unique individuals

The Canadian Community Health Survey (CCHS) is a national cross-sectional survey of the Canadian population that collects information related to health status, health care utilization, and health determinants. From 2000 to 2007 the survey was administered every 2 years to approximately 130,000 respondents; from 2007 onwards, data has been collected on an ongoing basis from 65,000 respondents per year and released annually with response rates declining over time (Fig. 1) [11–16]. The CCHS data are sampled from approximately 98% of the Canadian population aged 12 and older living in private dwellings within the 115 Heath Regions covering all provinces and territories. Individuals living on Indian Reserves and on Crown Land, institutional residents, full-time members of the Canadian Forces, and residents of certain remote regions are excluded.

Consent to record linkage and data sharing was obtained at time of survey (Fig. 1) and only those CCHS respondents who agreed were linked to death records and residential history through Statistics Canada’s Social Data Linkage Environment (SDLE) [17]. The linkage was approved by Statistics Canada [18] and is governed by the Directive on Microdata Linkage [19]. The linkage occurred within the Derived Record Depository, a highly secure linkage environment comprised of a national dynamic relational database of basic personal identifiers. Survey and administrative data are linked to the Derived Record Depository using a SAS-based generalized record linkage software (G-link) that supports deterministic and probabilistic linkage based on the mathematical theory of record linkage developed at Statistics Canada [20]. Mortality linkage to the Derived Record Depository between 2000 and 2016 was 99.8% [21]. A list of linked unique individuals is created through linkages that are deterministic (matching records based on unique identifiers) and probabilistic (matching records based on non-unique identifiers such as names, sex, date of birth, and postal code and estimating the likelihood that records are referring to the same entity). For the CCHS cycles considered, there was a linkage rate to the Derived Record Depository of 95.2% and a false error rate for the CCHS to SDLE linkage of 0.4% [22].

There were 666,000 CCHS respondents who agreed to data linkage (Fig. 1), reduced to 540,900 after excluding subjects with death dates prior to survey response dates (i.e. either the death record or linkage must be incorrect) or who were below the age of 25 or above 89 at time of survey as they are less likely to reside at the same postal code as their income tax mailing address [23]. The CCHS to SDLE linkage rates across key indicators were consistently high, ranging from 94.4% for the 20–29 age group to 96.2% for the 80–89 age group, 95.5 and 95.3% for males and females respectively, and by province/territory from 91.8% for the Yukon to 96.7% for Newfoundland and Labrador [22].

After linkage, we stacked the CCHS cycles into one data file. We standardized the variable categorizations when discrepancies between cycles existed. The covariates (listed, along with categorizations in Table 1) included socio-economic, behavioural, and contextual measures. More information about the definitions and classifications of these variables can be found elsewhere [9]. Provincially standardized deciles were calculated according to the distribution of residents in each province and the ratio of their total household income to the low-income cut-off for their corresponding household and community size. As this measure excluded subjects living in territories, we took the mean income within each decile and used these as cut-offs to categorize subjects living in territories by income into deciles. Once all subjects were placed in deciles, we merged groups to create quintiles.

**Table 1:** Descriptive statistics of the cohort and PM_2.5_ O_3_, and NO_2_ exposure, with Cox proportional hazard ratios

			95% CI							
Covariate	Person-years^a^	HR	Lower	Upper	PM_2.5_	S.D.	O_3_	S.D.	NO_2_	S.D.
All	4,452,700	–	–	–	5.9	2.0	36.0	7.5	8.6	5.9
Sex										
Male	1,995,100	–	–	–	5.9	2.0	35.9	7.6	8.5	5.9
Female	2,457,600	–	–	–	6.0	2.0	36.0	7.5	8.6	5.9
Age group (years)										
25 to 29	381,100	–	–	–	5.9	2.0	35.7	7.6	8.8	5.9
30 to 39	869,300	–	–	–	5.9	2.0	35.9	7.7	8.7	5.9
40 to 49	887,000	–	–	–	5.9	2.0	35.8	7.6	8.7	6.0
50 to 59	918,100	–	–	–	5.8	2.0	35.8	7.4	8.2	5.7
60 to 69	753,800	–	–	–	5.9	2.0	36.2	7.3	8.3	5.8
70 to 79	497,100	–	–	–	6.1	2.1	36.4	7.4	8.9	6.1
80 to 89	146,200	–	–	–	6.2	2.1	36.3	7.6	9.6	6.3
Immigrant status										
Non-immigrants	3,945,800	1.00	–	–	5.8	2.0	35.7	7.4	8.1	5.6
In Canada for 30+ years	317,900	0.86	0.83	0.88	6.8	2.1	38.5	8.0	11.2	6.5
In Canada for 20–29 years	92,300	0.74	0.68	0.80	7.0	2.0	37.4	8.0	13.2	7.0
In Canada for 10–19 years	96,800	0.62	0.56	0.69	7.2	1.9	37.6	7.7	14.5	7.0
Visible minority identity										
Not a visible minority	4,119,200	1.00	–	–	5.9	2.0	36.1	7.4	8.4	5.7
Visible Minority	244,900	0.89	0.84	0.93	6.6	2.1	34.9	8.9	12.8	7.2
Missing (dummy variable)	88,600	1.48	1.37	1.59	4.9	1.6	31.7	8.3	6.5	4.3
Indigenous identity										
Non-Indigenous or not stated	4,295,500	1.00	–	–	6.0	2.0	36.2	7.4	8.6	5.9
Indigenous	146,000	1.58	1.50	1.67	4.9	1.7	30.3	8.8	6.9	4.7
Missing (dummy variable)	11,300	1.16	0.99	1.35	5.6	1.8	35.0	7.5	8.2	5.6
Marital status										
Married or Common-law	2,823,000	1.00	–	–	5.8	2.0	36.1	7.6	8.1	5.5
Separated, Widowed, or Divorced	945,100	1.42	1.39	1.45	6.1	2.1	36.1	7.5	9.1	6.2
Single	681,400	1.57	1.52	1.62	6.2	2.1	35.4	7.5	9.8	6.8
Missing (dummy variable)	3200	1.58	1.20	2.07	5.9	1.9	34.5	8.0	9.4	6.1
Educational attainment										
No high school diploma	980,900	1.00	–	–	5.7	2.1	35.2	7.5	7.8	5.8
High School	757,200	0.82	0.80	0.84	6.0	2.0	36.6	7.7	8.6	5.8
Any post-secondary	1,926,400	0.77	0.75	0.78	5.9	2.0	36.0	7.5	8.4	5.7
University	752,100	0.55	0.54	0.57	6.2	2.0	36.4	7.4	10.1	6.5
Missing (dummy variable)	36,000	0.98	0.91	1.06	5.9	2.0	35.6	7.7	8.8	5.8
Employment status										
Employed	2,701,800	1.00	–	–	5.9	2.0	36.0	7.6	8.7	5.8
Unemployed	115,100	1.67	1.54	1.82	5.8	2.1	34.9	7.7	8.4	6.3
Not in work force	1,630,100	2.02	1.96	2.08	6.0	2.1	36.0	7.4	8.5	6.0
Missing (dummy variable)	5800	1.62	1.24	2.12	5.5	1.8	34.0	7.2	8.8	5.4
Income quintile										
Q1 (lowest income)	788,200	1.00	–	–	6.1	2.1	35.6	7.4	9.2	6.5
Q2	788,700	0.76	0.74	0.78	6.0	2.0	36.1	7.4	8.7	6.1
Q3	808,500	0.64	0.62	0.66	6.0	2.0	36.3	7.4	8.5	5.8
Q4	828,500	0.53	0.52	0.55	5.9	2.0	36.2	7.5	8.4	5.7
Q5 (highest income)	909,000	0.44	0.43	0.46	5.7	1.9	35.9	7.7	8.2	5.5
Missing (dummy variable)	329,800	0.74	0.72	0.77	5.8	2.0	35.5	8.0	8.6	5.8
Alcohol consumption										
Never drinker	392,400	1.00	–	–	5.8	2.0	35.8	7.3	8.0	5.9
Occasional drinker	840,300	0.84	0.81	0.86	5.8	2.0	35.7	7.6	8.3	5.8
Regular drinker, binging unknown	1,332,300	0.64	0.62	0.66	6.1	2.0	36.7	7.3	9.1	6.0
Regular, non-binge drinker	1,169,600	0.69	0.67	0.72	5.8	2.0	35.8	7.5	8.4	5.7
Regular, binge drinker	260,400	1.08	1.03	1.14	5.9	2.0	35.8	7.8	8.1	5.8
Former drinker	447,500	1.10	1.07	1.14	5.9	2.1	35.3	8.0	8.8	6.2
Missing (dummy variable)	10,200	0.93	0.81	1.07	5.6	1.9	34.8	7.5	8.5	5.5
Smoking behaviours										
Never smoker	1,293,700	1.00	–	–	6.0	2.0	36.4	7.5	9.0	6.1
Occasional smoker	177,200	2.11	2.00	2.23	6.0	2.0	35.6	7.7	9.0	6.3
Smoke under 10 cigarettes/day	263,000	2.45	2.35	2.55	5.9	2.1	35.3	7.9	8.7	6.2
Smoke 11–20 cigarettes /day	398,900	2.76	2.66	2.86	5.9	2.0	35.6	7.7	8.3	5.8
Smoke 20+ cigarettes /day	255,300	3.69	3.55	3.82	5.9	2.1	36.0	7.6	8.2	5.9
Former smoker	2,058,700	1.32	1.29	1.35	5.9	2.0	35.9	7.4	8.4	5.8
Missing (dummy variable)	6100	1.51	1.26	1.80	5.7	1.8	35.6	7.4	8.3	5.7
Fruit and vegetable consumption										
Under 5 servings/day	2,411,900	1.00	–	–	5.9	2.0	36.0	7.7	8.6	5.9
5–10 servings/day	1,450,300	0.82	0.80	0.83	6.0	2.0	36.6	7.5	8.8	5.9
10+ servings/day	132,700	0.82	0.77	0.87	6.1	2.0	36.6	7.5	8.9	6.0
Missing (dummy variable)	457,900	1.19	1.16	1.23	5.6	1.9	33.6	5.8	7.9	6.1
Leisure exercise frequency										
Active	1,005,700	1.00	–	–	5.9	2.0	36.1	7.6	8.6	5.7
Moderate	1,123,600	1.10	1.07	1.14	5.9	2.0	36.1	7.5	8.6	5.8
Inactive	2,224,700	1.70	1.65	1.74	5.9	2.0	35.9	7.5	8.5	6.0
Missing (dummy variable)	98,700	2.49	2.39	2.60	5.9	2.1	35.2	7.8	9.0	6.2
Body mass index (BMI)										
Normal weight (18.5–24.9)	1,425,400	1.00	–	–	6.1	2.0	36.1	7.4	9.1	6.2
Overweight (25.0–29.9)	1,671,700	0.81	0.80	0.83	5.9	2.0	36.0	7.5	8.5	5.8
Obese 1 (30.0–34.9)	800,500	0.93	0.90	0.95	5.8	2.0	35.9	7.6	8.1	5.6
Obese 2 (≥ 35)	355,300	1.33	1.28	1.37	5.7	2.0	35.7	7.7	7.9	5.5
Underweight (< 18.5)	57,700	2.13	2.00	2.26	6.2	2.1	36.0	7.4	9.7	6.5
Missing	142,200	1.61	1.53	1.68	5.8	2.0	35.8	7.8	8.2	5.5
Residential Instability										
Q1 (lowest marginalization)	993,500	1.00	–	–	5.3	1.8	36.9	7.8	7.1	4.8
Q2	1,231,300	0.98	0.95	1.00	5.6	1.9	36.7	7.9	7.1	4.8
Q3	957,500	0.98	0.95	1.01	5.9	2.0	34.6	7.7	8.6	5.5
Q4	780,100	0.96	0.93	0.99	6.5	2.0	35.7	6.8	10.0	6.2
Q5 (highest marginalization)	490,300	1.04	1.01	1.07	7.2	1.9	35.5	6.1	13.0	7.6
Dependency										
Q1 (lowest marginalization)	701,400	1.00	–	–	5.7	1.9	33.4	8.7	10.0	6.2
Q2	601,900	0.96	0.92	0.99	6.1	1.9	35.8	7.3	9.9	6.0
Q3	602,400	0.92	0.89	0.95	6.3	2.1	37.4	7.5	9.7	6.1
Q4	945,800	0.90	0.87	0.93	6.2	2.1	37.6	7.5	8.6	5.8
Q5 (highest marginalization)	1,601,300	0.88	0.86	0.91	5.7	2.0	35.7	6.7	7.0	5.3
Material deprivation										
Q1 (lowest marginalization)	713,600	1.00	–	–	6.0	1.8	37.7	7.4	9.8	5.2
Q2	777,700	1.03	1.00	1.06	6.2	1.9	38.0	8.0	9.4	5.4
Q3	897,400	1.04	1.01	1.07	6.2	1.9	37.5	7.3	8.8	5.5
Q4	783,800	1.08	1.04	1.11	6.2	2.1	35.7	7.5	9.2	6.3
Q5 (highest marginalization)	1,280,200	1.15	1.12	1.19	5.3	2.0	32.9	6.4	6.8	6.2
Ethnic concentration										
Q1 (lowest marginalization)	1,839,100	1.00	–	–	5.2	1.7	35.7	6.9	5.9	3.8
Q2	1,211,600	1.02	1.00	1.04	6.0	2.0	36.7	8.0	8.0	4.8
Q3	749,900	1.01	0.99	1.04	6.3	2.0	35.5	7.8	10.1	5.7
Q4	409,500	1.04	1.00	1.07	7.0	2.1	35.7	8.2	14.2	6.9
Q5 (highest marginalization)	242,600	0.98	0.94	1.02	7.7	1.7	36.4	7.5	17.7	6.2
Census Metropolitan Area/Census Agglomeration size										
Not applicable (non-CMA/CA)	1,485,900	1.00	–	–	4.7	1.3	33.9	7.1	4.9	2.7
10,000–29,999	355,900	1.03	0.99	1.06	5.0	1.3	31.6	7.9	6.0	3.1
30,000–99,999	570,900	1.03	1.00	1.06	5.8	1.8	36.6	6.9	7.1	3.3
100,000–499,999	872,600	1.00	0.98	1.03	6.8	2.2	39.5	8.0	8.9	4.6
500,000-1,499,999	506,400	0.94	0.91	0.97	6.7	1.7	36.5	6.2	13.2	6.2
> 1,500,000	661,000	0.91	0.89	0.94	7.5	1.7	37.4	6.5	15.5	6.9
Urban form										
Active urban core	304,800	1.00	–	–	7.6	1.9	36.7	7.1	14.5	7.1
Transit-reliant suburb	179,500	0.98	0.93	1.04	7.8	1.7	36.7	7.1	16.1	7.4
Car-reliant suburb	1,242,700	0.81	0.78	0.84	7.0	1.9	38.3	7.0	12.1	6.0
Exurban	216,400	0.83	0.78	0.87	5.5	1.6	38.7	7.0	6.8	3.6
Non-CMA/CA	2,509,400	0.93	0.90	0.96	5.1	1.6	34.4	7.5	5.7	3.2
Airshed										
East Central	2,041,500	1.00	–	–	7.0	2.1	41.3	6.0	9.9	6.6
Northern	117,800	1.18	1.11	1.27	3.9	1.2	26.0	7.0	4.7	2.6
Southern Atlantic	711,700	1.11	1.08	1.13	4.4	1.0	30.7	3.1	3.9	2.6
Prairie	666,900	1.00	0.97	1.02	5.2	1.4	34.1	5.7	9.5	4.9
West Central	397,100	1.07	1.03	1.10	4.9	1.1	31.3	5.0	8.0	4.9
Western	517,600	1.04	1.01	1.07	5.7	1.5	30.6	5.9	9.8	5.0

Columns 6, 8, and 10 are mean values

^a^numbers rounded to the nearest 100 for confidentiality

Postal code history was complete from 1981 to 2016 for 35.0% of respondents and 12.6% of respondents had no postal code history. There were gaps in postal code histories for 52.4% of respondents, which is to be expected, as taxes may not have been filed for various reasons (e.g immigration, death, or age). We imputed complete or partial postal codes only when bookended by postal codes with sufficient similarity before and after the gap [24]. For example, if a postal code in 2008 was K1A 0T6 and then K1A 0K9 in 2012, a partial postal code of K1A 0** would be imputed for the four missing years from 2009 to 2011. We did not impute postal codes if a gap existed at the beginning or end of the follow-up period or after a person’s death; full or partial postal codes (two to five digits) were imputed for 1.5% of person-years.

We organised the cohort into a person-year file with each year of exposure (1981–2016) per person representing a row of data. Subsequently we excluded specific person-years [1] once they turned age 90 during follow-up [2], if the person had immigrated to Canada less than 10 years prior to survey interview [3], if there was no postal code [4], if the postal code could not be linked to air pollution or contextual covariates [5], if the PM_2.5_ three-year moving average with a one-year lag was calculated by fewer than 2 years of exposure data, or [6] if the person-year was before survey interview date or after a person’s death (Fig. 1). We excluded recent immigrants to Canada (10 years or less) since they have spent the majority of their lives outside of Canada with unknown exposure, and this time exceeds the number of years in Canada where exposure can be estimated.

### Exposure file and analytical file

The task of linking contextual covariates and air pollution values to the cohort required the creation of a master list of postal codes with their respective points of latitude and longitude and census geography. We produced this list from Statistics Canada’s June 2017 Postal Code Conversion File and the two previous versions (August 2015 and May 2011) to ensure coverage of retired postal codes [25–27]. Since census geography does not align with postal code locations, a single postal code can have multiple points of latitude and longitude. Each can represent the centroid of a blockface (i.e. a street block), dissemination block (i.e. an area bounded on all sides by roads), or dissemination area (i.e. adjacent dissemination blocks that collectively contain 400 to 700 persons) within a postal code.

We developed and used annual exposure estimates of PM_2.5_ from 1998 to 2012 by relating satellite retrievals of aerosol optical depth (AOD) to near-surface PM_2.5_ concentrations using the geophysically-based relationship simulated by a chemical transport model [28]. Ground monitoring data from the National Air Pollution Surveillance (NAPS) network were then incorporated, along with other North American-based measurements, to constrain these PM_2.5_ estimates with geographically weighted regression. The resulting ambient PM_2.5_ surface provided estimates for North America at about a 1km^2^ resolution [28]. Spatial variation from this surface was used with simulated PM_2.5_ and consistently constrained with local ground-based monitors to extend our PM_2.5_ coverage to 2015 [29].

The ambient warm season daily-maximum eight-hour average O_3_ surfaces were developed by Environment and Climate Change Canada for 2002–2015 using chemical transport modelling informed by surface observations as hourly estimates from 2002 to 2015 [30–32]. Estimates of NO_2_ were created using a national land use regression model (LUR) informed by on satellite-derived NO_2_ (10 km resolution), distances to highways and major roads, and roadway kernel density gradients [33]. Ozone and NO_2_ values were back-casted to obtain exposures for 1981–2015 using ground monitoring data from the Canadian National Air Pollution Surveillance program. Annual adjustment factors were calculated at a census division level from the ratio of observed concentration to the values in the surface for the reference year (see Pinault et al. for more detail [9]).

We linked postal codes to PM_2.5_ in ArcGIS Desktop 10.5.1 using the points of latitude and longitude from the master postal code list and the air pollutant surfaces. In cases where there were multiple points of latitude and longitude for a single urban postal code, we used equal weighting of the multiple air pollutant values to provide a singular value. In rural communities, we took the population-weighted average of the values associated with duplicate postal codes. We used population-weighing to average multiple values to create inputs for partial postal codes (2 to 5 digits).

### Covariates

Contextual covariates were available at various census geographies and we merged these to individual person-years via postal codes (as described below). We created historic measures when possible to reflect neighbourhood-level changes over the time.

Regions of Canada that share air quality characteristics and movement patterns have been defined by the Air Quality Management System (AQMS) as six distinct airsheds [34]. By subdividing the country into large geographic areas, adjustment for the broad spatial variation in mortality rates can be performed [9, 34]. We assigned airshed to the cohort by postal code. We used a population weighted mode in cases where there were multiple points of latitude and longitude for a single postal code.

We developed a historic community size variable to account for different sizes of metropolitan regions and changes in population over time, classifying Census Metropolitan Areas (CMAs: major urban core, 100,000+ residents) and Census Agglomerations (CAs: smaller urban cores, 10,000+ residents) by population counts [35]. Since CMA/CAs cover large areas that can include farmland near the urban-rural fringe and residential enclaves of commuters to the city, we created a measure that accounts for differences in urban form within these CMA/CAs. We used population density measures (1991–2016) and frequencies for different modes of transportation at the neighbourhood level (1996–2016) to categorize census tracts as active urban core, transit-reliant suburb, car-reliant suburb, exurban, and non-CMA/CA [36]. Both CMA/CA size and urban form were attached to the postal code list via census geography before merging with the cohort. In cases where there were multiple points of latitude and longitude representing a postal code, we used a population-weighted mode to assign categories.

The Canadian Marginalization Index (Can-MARG) is a measure of community-level marginalization comprised of four factors: material deprivation (e.g. proportion of people living in dwellings in need of repair), residential instability (e.g. proportion of people who live in a dwelling that they do not own) dependency (e.g. proportion of seniors and youth compared to those who are not), and ethnic concentration (e.g. proportion of recent immigrants and self-reported visible minorities) [37]. We used historic census tract-level Can-MARG values in CMA/CAs, and a population-weighted aggregation of the dissemination area-level Can-MARG values at the census subdivision level in rural areas outside of CMA/CAs that are not covered by census tracts. We assigned Can-MARG values to points of latitude and longitude before quintiles were assigned.

### Statistical analysis

We calculated for each individual and year of follow-up a three-year moving average for PM_2.5_ with a one-year lag, (e.g. the exposure in 2002 is the average of exposures in 1999, 2000, and 2001).

We ran standard Cox proportional hazard models to assess the relationship between PM_2.5_ exposure and non-accidental death (ICD-10 codes A to R) from survey interview year to the end of follow-up period or year of death. We started model building with a baseline hazard function for PM_2.5_ stratified by five-year age groups, sex, and survey cycle to ensure that respondents within these strata would be broadly comparable. We calculated new hazard ratios for models that included each socio-economic and behavioural covariate individually. We included covariates in the partially-adjusted model if the log difference between the new hazard ratio and the baseline was more than 10%. Subsequently, we added contextual covariates individually to the partially-adjusted model and included them in the final model using the same criteria (comparing to the partially-adjusted model that included socio-economic and behavioural covariates). All covariates considered for inclusion in the final model and the associated hazard ratios are found in Table 1.

We examined the shape of the association between PM_2.5_ and mortality with a SCHIF [10]. This method is based on a construction of several transformations of concentration and fitting the transformed variable in a Cox model, estimating the log-hazard ratio for a unit change in the transformed variable and its standard error. An ensemble of all models examined was then constructed using a weighted average of the predicted log-hazard ratio and any concentration, with weights defined by the AIC of each model. The transformations are variations on a sigmoidal function which yields supra-linear, near linear, and sub-linear shapes.

### Sensitivity analyses

We examined effect modification by select socio-economic and behavioural covariates, and by high- and low-exposure groups to the combined oxidant capacity of NO_2_ and O_3_ (henceforth: O_X_) which is calculated as the redox-weighted oxidant capacity [38] i.e. a weighted average using redox potentials as the weights (O_x_^wt^ = [(1.07 V × NO_2_) + (2.075 V × O_3_)]/3.145 V) (Table 4). We examined multiple pollutant models to investigate whether the inclusion of other common pollutants (NO_2_, O_3_, and O_X_) in the model may modify the PM_2.5_-mortality relationship [5, 39].

## Results

There were 4,452,700 person-years in the cohort after exclusion criteria were applied (Fig. 1) from 452,700 unique individuals. Entry into the cohort and length of the follow-up period varied by survey cycle, with the first cohort having up to 15 years of follow-up. For those who died, the average follow-up period was 5.1 years (s.d. 3.4); it was 6.5 years (s.d. 4.1) for those who survived the follow-up period. There were 50,700 non-accidental deaths. Of these, there were 7900 deaths from ischemic heart disease, 2800 from cerebrovascular disease, and 4300 from other cardiovascular diseases; 900 from pneumonia, 2800 from COPD, and 1100 from other respiratory diseases; 5500 from lung cancer, 1300 from colon cancer, 1300 from breast cancer, 1100 from pancreatic cancer, and 9900 from all other cancers. Further, there were 1700 deaths from diabetes, 3900 deaths from neuropsychiatric conditions, 2200 from digestive diseases, 1100 from genitourinary diseases and 3000 from all other non-accidental causes.

Exposure to PM_2.5_ was higher in women, more recent immigrants, and non-Indigenous people. Being single, university educated, and in the poorest income quintile were also associated with higher exposures (Table 1). We observed higher exposure to PM_2.5_ in people living in the largest CMAs and in the East Central airshed (which includes Toronto and Montreal). The distribution of exposure estimates for PM_2.5_, NO_2_, O_3_, and O_X_ is found in Table 2.

**Table 2:** Distribution of air pollutant values for all person-years

	mean	minimum	percentile					maximum
			5th	25th	50th	75th	95th	
PM_2.5_	5.9	0.4	3.4	4.3	5.5	7.1	9.7	17.2
O_3_	36.0	3.1	24.9	30.7	35.3	40.9	49.0	65.8
NO_2_	8.6	0.0	2.3	4.4	6.9	11.1	20.5	69.1
O_X_^a^	26.7	4.1	18.6	22.6	26.2	30.6	36.5	54.1

^a^the combined oxidant capacity of NO_2_ and O_3_

The cohort was generally representative of the Canadian population, as seen through their mortality rates by subgroup (Table 1). Immigrants and non-Indigenous people had lower mortality rates compared to their counterparts. Being married, holding a university degree, and being employed were associated with a lower risk of mortality. As expected, there were clear trends in mortality risk with income, education, and immigrant status.

The unadjusted model had a hazard ratio of 0.96 (95% CI 0.92–1.00) which increased to 1.11 (95% CI 1.04–1.18) when adjusted by the socio-economic, behavioural, and contextual covariates that met the inclusion threshold (Table 3). All covariates except for body mass index (BMI), employment status, and urban form met the criteria and were included in the final model. When we added the behavioural covariates to a model that included only socio-economic covariates the hazard ratio increased from 1.05 (95% CI 1.00–1.09) to 1.09 (95% CI 1.05–1.15). Conversely, when we added the behavioural covariates to a model that included both the socio-economic and contextual covariates, they lowered the PM_2.5_ hazard ratio from 1.13 (95% CI 1.06–1.21) to 1.11 (95% CI 1.04–1.18).

**Table 3:** Cox proportional hazard ratios for non-accidental mortality^a^ and PM_2.5_ exposure, all respondents, 10% inclusion threshold

Model		95% CI		
	HR	Lower	Upper	-2 LL
Unadjusted (stratified by age, sex, and cycle)	0.96	0.92	1.00	769,047.51
Socio-economic covariates (Unadjusted model +)				
Visible minority identity	0.98	0.93	1.02	768,923.2
Indigenous identity	0.98	0.94	1.03	768,812.8
Immigrant status	1.02	0.98	1.07	768,784.2
Educational attainment	1.05	1.01	1.10	767,553.7
Marital status	0.92	0.88	0.96	767,479.4
Income quintile	0.94	0.90	0.98	766,080.2
Adjusted by socio-economic covariates*	1.05	1.00	1.09	764,396.4
Behavioural level covariates (Unadjusted model +)				
Fruit and vegetable consumption	1.00	0.96	1.05	768,304.6
Leisure exercise frequency	1.00	0.96	1.05	768,304.6
Alcohol consumption	1.04	1.00	1.09	766,726.1
Smoking behaviours	0.97	0.93	1.02	762,432.6
Adjusted by all socio-economic + behavioural covariates	1.09	1.05	1.15	756,074.0
Contextual covariates (Adjusted by socio-economic covariates +)				
Ethnic concentration	1.00	0.95	1.05	764,411.6
Material deprivation	1.04	0.99	1.09	764,411.2
Residential instability	1.06	1.01	1.11	764,408.0
Census Metropolitan Area/Census Agglomeration size	1.04	0.98	1.09	764,401.7
Airshed	1.11	1.05	1.17	764,378.8
Dependency	1.03	0.98	1.08	764,314.1
Adjusted by all socio-economic + contextual covariates*	1.13	1.06	1.21	764,157.5
Contextual covariates (Adjusted by socio-economic + behavioural covariates +)				
Ethnic concentration	1.05	1.00	1.10	756,050.7
Material deprivation	1.12	1.07	1.17	756,049.0
Census Metropolitan Area/Census Agglomeration size	1.05	0.99	1.10	756,039.5
Dependency	1.08	1.03	1.13	755,985.4
Airshed	1.11	1.05	1.17	755,969.1
Residential instability	1.08	1.03	1.13	755,962.9
Final model (Adjusted by all socio-economic + behavioural + contextual covariates)	1.11	1.04	1.18	755,760.2

^a^due to a 10 μg/m^3^ increase in PM_2.5_ concentration

**Fig. 2:**
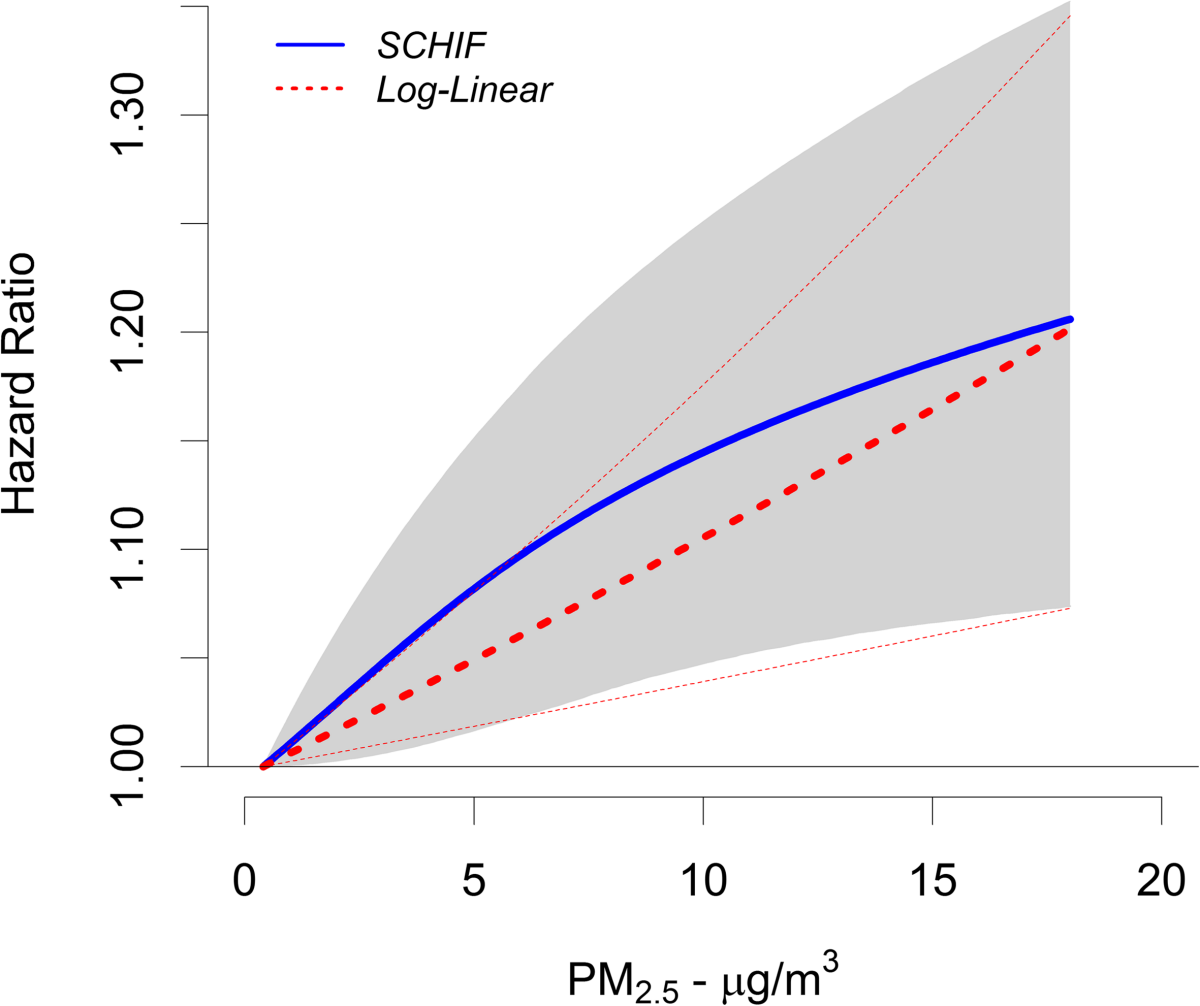
SCHIF model of PM_2.5_

The SCHIF characterisation of the PM_2.5_-mortality association (for all cohort members) displayed a supra-linear shape that rises in a steeper fashion compared to the standard log-linear model prediction for lower concentrations and changes in a more moderate manner for higher levels (Fig. 2). Note that the SCHIF displays wider uncertainty intervals compared to the log-linear model at low concentrations, in part due to the additional variation associated with model shape, a feature captured by the SCHIF but not the log-linear model. We observed a positive and statistically significant (*p* < 0.05) association between PM_2.5_ and non-accidental mortality for all concentrations examined as indicated by the SCHIF hazard ratio predictions.

We assessed effect modification within the PM_2.5_-mortality relationship by separating the cohort by age, sex, immigrant status (i.e. immigrants who had been in Canada for 10 or more years vs. non-immigrants), and educational attainment, and comparing resulting hazard ratios with Cochrane’s Q (Table 4). The hazard ratio was 4% higher for males (1.13 95% CI 1.03–1.23) than females (1.09 95% CI 0.99–1.19). When contrasted by age, the hazard ratio was 9% lower for those aged 75 years or more (1.04 95% CI 0.94–1.16) compared to those aged 65–74 (1.13 95% CI 1.01–1.27) and 10% lower compared to those aged 65 or less (1.14 95% CI 1.01–1.29). The hazard ratio for non-immigrants was higher than that of the final model (1.14 95% CI 1.07–1.23) and the immigrant group had a null hazard ratio (0.98 95% CI 0.83–1.16). The hazard ratio for those without a high school diploma (1.08 95% CI 098–1.19) was lower than those who graduated from high school (1.14 95% CI 1.04–1.24). The Cochrane’s Q *p*-values did not indicate that the above hazard ratios were significantly different between subgroups. We repeated the effect modification analyses for behavioural covariates. There was no significant difference between those who consumed fewer than five servings of fruits and vegetables per day compared to those who consumed five or more (1.10 95% CI 1.01–1.20 vs. 1.16 95% CI 1.04–1.30) although the hazard ratio was higher for those who consumed more fruits and vegetables. We found that hazard ratios were higher for regular drinkers (1.18 95% CI 1.09–1.28) and daily or occasional smokers (1.13 95% CI 0.99–1.27) compared to never or former drinkers (1.01 95% CI 0.90–1.12) or never or former smokers (1.11 95% CI 1.03–1.20), with a significant difference found between those who do and do not consume alcohol (*p* < 0.05). The HRs produced for each subgroup were pooled (Table 4), resulting in HRs that were similar to the full cohort final model. The high- and low- O_x_ groups had significantly different PM_2.5_-mortality hazard ratios. The inclusion of other pollutants (O_3_, NO_2_, and O_X_) attenuated the PM_2.5_ hazard ratios and produced confidence intervals that include a null value, with the greatest reduction seen in the model that included PM_2.5_, NO_2_, and O_3_ (1.00 95% CI 0.98–1.02, 1.03 95% CI 1.01–1.05, 1.05 95% CI 1.03–1.07 respectively) (Table 5).

**Table 4:** Examination of effect modification for non-accidental mortality^a^ for the cohort through Cox proportional hazards models and Cochrane’s Q

		95% CI	
	HR	Lower	Upper
Full cohort^b^	1.11	1.04	1.18
Sex			
Male	1.13	1.03	1.23
Female	1.09	0.99	1.19
Pooled HR	1.11	1.04	1.18
Age			
Under 65	1.14	1.01	1.29
65–74	1.13	1.01	1.27
75 or over	1.04	0.94	1.16
Pooled HR	1.10	1.03	1.17
Immigrant status			
Non-immigrants	1.14	1.07	1.23
Immigrants^b^	0.98	0.83	1.16
Pooled HR	1.12	1.05	1.19
Educational attainment			
No high school	1.08	0.98	1.19
High school graduate	1.14	1.04	1.24
Pooled HR	1.11	1.04	1.19
Fruit and vegetable consumption (servings per day)			
Less than five	1.10	1.01	1.20
Five or more	1.16	1.04	1.30
Pooled HR	1.12	1.05	1.20
Alcohol consumption			
Never or former drinker	1.01*	0.90	1.12
Occasional or regular drinker	1.18*	1.09	1.28
Pooled HR	1.12	1.05	1.19
Smoking behaviours			
Never or former smoker	1.11	1.03	1.20
Daily or occasional smoker	1.13	0.99	1.27
Pooled HR	1.12	1.05	1.19
Oxidant capacity^c^			
Low O_X_	0.92*	0.81	1.04
High O_X_	1.16*	1.07	1.26
Pooled HR	1.08	1.01	1.16

^a^due to a 10 μg/m^3^ increase in PM_2.5_ concentration

^b^excludes immigrants who have been living in Canada for fewer than ten years

^c^above or below the median value of Oxidant Capacity of all person-years (26.19 ppb)

^*^Cochrane’s Q *p*<0.05

**Table 5:** Cox proportional hazard ratios for non-accidental mortality^a^ and PM_2.5_ NO_2_, O_3_, and O_X_, and multiple-pollutant models

			95% CI				
	Pollutant	HR	Lower	Upper	-2 LL	SBC	AIC
PM_2.5_	PM_2.5_	1.03	1.01	1.05	755,760.2	756,453.5	755,888.2
O_3_	O_3_	1.05	1.03	1.07	755,742.1	756,435.4	755,870.1
NO_2_	NO_2_	1.03	1.02	1.05	755,756.9	756,450.3	755,884.9
O_X_^b^	O_X_	1.06	1.04	1.09	755,734.0	756,427.3	755,862.0
PM_2.5_ and O_3_	PM_2.5_	1.01	1.00	1.03			
	O_3_	1.05	1.03	1.07	755,740.0	756,444.2	755,870.0
PM_2.5_ and NO_2_	PM_2.5_	1.02	1.00	1.04			
	NO_2_	1.03	1.01	1.05	755,753.4	756,457.6	755,883.4
PM_2.5_ and O_X_^b^	PM_2.5_	1.01	0.99	1.03			
	O_X_	1.06	1.04	1.09	755,733.7	756,437.9	755,863.7
PM_2.5_, O_3_ and NO_2_	PM_2.5_	1.00	0.98	1.02			
	O_3_	1.05	1.03	1.07			
	NO_2_	1.03	1.01	1.05	755,732.5	756,447.5	755,864.5

^a^hazard ratios are per increase in inter-quartile range: PM_2.5_ 2.80 μg/m^3^, O_3_ 10.20 ppb, NO_2_ 6.63 ppb, O_X_ 8.05 ppb

^b^the combined oxidant capacity of NO_2_ and O_3_

## Discussion

Using a cohort comprised of several cycles of a health survey with up to a 15-year follow-up period and high resolution exposure estimates, we found that exposure to PM_2.5_ was associated with an 11% increase in non-accidental mortality per 10 μg/m^3^ after extensive adjustment for socio-economic, behavioural, and contextual covariates.

The hazard ratio for the full cohort was similar to that of the Nurse’s Health Study in the United States (1.13 95% CI 1.05–1.22) that adjusted for individual-level socio-economic and behavioural covariates [40] and a cohort from England (1.13 95% CI 1.00–1.25) that controlled for smoking, BMI, income, age, and sex [41]. Burnett and colleagues [42] report hazard ratio estimates for a 10 μg/m^3^ change in long-term exposure to PM_2.5_ and non-accidental mortality in 41 cohorts conducted globally, 36 of which included adjustment for behavioural risk factors. The pooled hazard ratio among these 36 cohorts was 1.09 (95% CI 1.05–1.12), a value similar to that observed in our current study (1.11 95% CI 1.04–1.18). A version of the 2001 CanCHEC census-based cohort produced a hazard ratio that is similar to this work (1.09 95% CI 1.07–1.11) [6].

The impact of individual-level behavioural risk factors on the PM_2.5_-mortality association was assessed to address a common critique of many large administrative cohort studies examining the air pollution-mortality relationship. The inclusion of behavioural covariates to a model including socio-economic and ecological covariates lowered the PM_2.5_ hazard ratio 2% (from 1.13 to 1.11). This modest change in the hazard ratio can be interpreted to indicate that the behavioural covariates were being adequately controlled for by the socio-economic and ecological covariates in the established relationship between PM_2.5_ exposure and non-accidental mortality. This finding is similar to the previous CCHS cohort analysis and analysis of a Medicare-based cohort; both reported that adjustment for behavioural covariates had a minimal effect on hazard ratios [3, 7]. There is evidence (Tables 3 and 4) for a small increase in risk of PM_2.5_-related mortality in occasional or regular drinkers but this may be masked by null effects from the inclusion of other behavioural covariates (fruit and vegetable consumption, smoking behaviours) and this confounding is likely the result of the spatial distribution of drinking behaviours, with binge drinkers having the largest mortality risk but lower PM_2.5_ exposures. This study, through its inclusion of multiple covariates and an explicit a priori analysis approach for model building therefore provides the most extensive evidence to date that, in the Canadian context, missing data on behavioural risk factors for mortality have a minimal confounding bias on the PM_2.5_-mortality association.

The increase in the PM_2.5_ hazard ratio with the addition of the ecological covariates was largely driven by the addition of airsheds. Not only do these airsheds characterize broad air movement patterns, they also capture areas with similar composition of PM_2.5_ (e.g., proportion of PM_2.5_ composed of nitrate is highest in the Prairie airshed, whereas the Southern Atlantic airshed is composed of a notably higher proportion of black carbon) [34]. They also delineate general socio-cultural groups with distinct mortality risk factors beyond those captured by the typical socioeconomic census variables included in our survival models. The three airsheds with the largest hazard ratios, along with high material deprivation, all have the lowest levels of air pollutants which would account for the negative confounding effect observed in Table 3. Further, the largest airshed (East Central) contains both Toronto and Montreal, the two largest CMAs in Canada and significant population hubs. High PM_2.5_ exposure and related mortality are largely driven by the population of Toronto (21% of the national population in 2006) where the mean PM_2.5_ exposure is 9.33 μg/m^3^ whereas the mean in the rest of the country is 7.68 μg/m^3^ [43]. These results are consistent with a descriptive analysis of PM_2.5_ exposure in 2006 long-form census respondents [9]. Although urban areas are the most common residence for both high income and highly educated Canadians, rural residences are more common among the high income earners than university graduates (i.e within the highest income quintile, 73.7% urban vs 26.3% urban fringe or rural; among those who are university educated, 82.6% urban vs. 17.3% urban fringe or rural). The greater tendency for high-income Canadians to live in rural areas is consistent with the findings in this paper. As a result, PM_2.5_ exposure by income categories is a slightly more linear pattern than education in both of these studies.

We estimated the shape of the concentration-response (CR) function for the PM_2.5_-mortality association. A slight supra-linear association (Fig. 2) was found, with a steep CR function at the low to median PM_2.5_ range which levelled off slightly after approximately 10 μg/m^3^. The SCHIF hazard ratio predictions indicated a positive and significant association between PM_2.5_ and non-accidental mortality for all concentrations, suggesting risks to concentrations below 2 μg/m^3^. Previous work using a CCHS-based cohort used a spline-based procedure and found that the shape of the relationship between non-accidental mortality and PM_2.5_ was supra-linear in shape with a threshold of 4.5 μg/m^3^, but was limited due to wide confidence intervals [9]. A study in China using a SCHIF function found non-linear relationships for multiple causes of death [44]. Such a relationship, when applied in a health impact framework, as in the Global Burden of Disease [45, 46] and in the recent Global Exposure Mortality Model [42] suggest benefits both from reducing PM_2.5_ concentrations areas with the highest concentrations and from continuing to reduce them in relatively cleaner areas, including Canada, where it is estimated that the entire population now lives in areas with ambient PM_2.5_ concentrations below the current WHO Guideline [47]. Worldwide it is estimated that small absolute reductions under 3 μg/m^3^ could prevent hundreds of thousands of deaths in areas that comparatively have low levels of PM_2.5_ [48].

The risk of non-accidental mortality from exposure to PM_2.5_ was 4% higher in males over females (males 1.13, females 1.09), a pattern that has emerged in similar work. The hazard ratios from the current study are more aligned with the ESCAPE European pooled cohort (males 1.14 95% CI 1.04–1.24; females 0.99 95% CI 0.92–1.07) [2] albeit with a higher hazard ratio for women when compared to the previous version of CCHS-based cohort (males 1.34 95% CI 1.24–1.46; females 1.18 95% CI 1.09–1.28) [9]. Hazard ratios were lowest for members of the cohort aged 75 and older (1.04) and were similar for those aged 65 and under (1.14) and 65 to 75 (1.13); this is similar to the European study which found that risk decreases with age (< 60 years 1.16 95% CI 1.00–1.34; 60–75 years 1.10 95% CI 1.00–1.20; ≥75 years 1.03 95% CI 0.95–1.11). When we divided the cohort into immigrants (in Canada for 10 years or more) and non-immigrants, the PM_2.5_-mortality association increased for non-immigrants and was null among the immigrant population. This result is consistent with prior Canadian census-based cohort studies [5] and is possibly the result of what is termed the “healthy immigrant effect” [49–53], likely intensified by the preferential settlement of immigrants into the largest cities which have higher PM_2.5_ exposure. The hazard ratio for high school graduates (1.14) was higher than for those without a diploma (1.08) which is to be expected given that the latter is more likely to live in rural areas [43], and have a mean PM_2.5_ exposure that is lower than other educational groups (Table 1) [43]. We examined effect modification by behavioural covariates (i.e., fruit and vegetable consumption, smoking behaviour, and alcohol consumption) and found significant difference in the resulting hazard ratios only in the case of alcohol consumption. Effect modification analyses on the ESCAPE cohort also found no effect modification by fruit and vegetable consumption or smoking behaviour, but did not consider alcohol consumption [2].

The multiple pollutant models indicated that the relationship between non-accidental mortality and PM_2.5_ exposure are attenuated when we included other pollutants (NO_2_, O_3_, and O_X_) in the models. These findings indicate both that PM_2.5_ is associated with mortality and that the inclusion of gaseous co-pollutants, O_x_ in particular, may better characterize the biologically active aspects of PM_2.5_ and the overall air pollution mixture compared to the PM_2.5_ mass concentration [5]. Weichenthal et al. looked at the effect modification of oxidant gases on PM_2.5_ more specifically and found that spatial variations in O_x_ concentrations may act as surrogates for the presence or absence of harmful air pollutant mixtures that enhance PM_2.5_ toxicity [42]. We examined the PM_2.5_-mortality association in both low- and high- O_x_ person-years and found a 24% difference in risk. Our findings support these previous studies using different longitudinal Canadian cohorts and that knowledge of interactions between PM_2.5_ and oxidant gases leading to adverse health will improve risk management activities and public health.

We performed this analysis on an extended and updated version of a cohort described in a previous study by Pinault et al. [9] with improvements to the exposure assessment and linkage to death, postal code history, environmental exposures, and contextual covariates. While some of the results are comparable to the previous cohort (e.g. socio-economic + behavioural covariate models are within a 1% margin), there are differences in the covariates included in the final models and the resulting hazard ratios. This is not unexpected since the contextual covariates addressing area-level marginalization in the two studies were created differently (area-level proportions of specific variables vs. a principle component analysis which resulted in four factors), and measured at different geographical units (census divisions vs. census tracts and census subdivisions). Another difference is that the updated cohort and current work includes immigrants who have lived in Canada for ten or more years whereas the previous work only included those who had been in Canada for 20 or more years. This newly included group of semi-recent immigrants (10–19 years in Canada) have substantially lower hazard ratios of mortality compared to the non-immigrant population (Table 1). Their inclusion in the current study acts to reduce the overall PM_2.5_ hazard ratio (Table 4).

This large, national cohort is an extension and improvement to the previous CCHS-Mortality cohort, with an updated linkage and extended follow-up period for mortality and postal code history which now spans 36 years (1981 to 2016). More broadly the cohort has many strengths, including the fine resolution of the PM_2.5_ estimates (1km^2^), the ability to incorporate mobility across the follow-up years, an explicit a priori model building strategy, the inclusion of multiple time-varying contextual covariates to address spatial, neighbourhood- and city-level characteristics, and most uniquely the behavioural covariates such as smoking behaviours, alcohol consumption, diet, and exercise to control for health behaviours related to mortality that are not typically found on cohorts of this size.

This cohort and the analysis are limited by the data available. First, postal code history was derived from tax and administrative data. Historical postal codes reflect the mailing address as reported on a tax return and not necessarily a person’s residence; in 92.9% of cases the postal code reflects the person’s residence at time of survey [23]. Similarly, outdoor ambient levels of PM_2.5_ at a person’s residence may not reflect their actual exposure. Sensitivity analysis performed with the 2001 CanCHEC found that finer scale resolution (1km^2^) estimates of PM_2.5_ resulted in lower AIC values and higher hazard ratios in the PM_2.5_-mortality model for non-accidental death compared to a 10km^2^ or 5km^2^ grid indicating that exposure estimates that are more specific to a person’s residence are appropriate [54]. Gaps in postal code history are imputed under the assumption that the person did not leave the country or community during that time. In assigning contextual covariates by postal code, misclassification may occur from taking the mode or mean when estimating a single value to represent multiple points of latitude and longitude for a single postal code. Second, in contrast to the CanCHEC cohorts (Pappin AJ, Crouse DL, Christidis T, Pinault LL, Tjepkema M, Erickson A, Brauer M, Weichenthal S, van Donkelaar A, Martin RV, Brook J, Hystad P. Burnett RT. Associations between low levels of fine particulate matter andmortality within Canadian cohorts. Environ Health Persp., under review), this cohort does not completely represent the full Canadian population; the Canadian Community Health Survey is not a census of the population and survey weights were not used in this analysis. Further, in creating this cohort persons were removed if they did not consent to data linkage or if they could not be linked to the SDLE. The CCHS over-samples rural communities [55] which results in a disproportionate sample in areas with low levels of PM_2.5_ and higher rates of mortality. The sampling framework and un-weighted analysis likely caused the null unadjusted hazard ratio which became positive as covariates were added to the model to address confounding. These results are consistent with the Agricultural Health study which examined non-accidental death related to PM_2.5_ in rural communities in two American states (Iowa and North Carolina) and found a protective hazard ratio in minimally and fully adjusted models [56]. Regardless, the protective unadjusted hazard ratio should not come as a surprise as contextual and socio-economic covariates are included in models because we know that they are related to both PM_2.5_ and mortality and can act as confounders (see Table 1 for the mortality Hazard Ratios by individual covariates). Given that these factors covary with both mortality and PM_2.5_ their inclusion in the models is crucial. We suggest that the unadjusted model is not reflective of the PM_2.5_-mortality relationship and that the direction or magnitude should not be over-interpreted. Third, although this cohort includes behavioural covariates these are self-reported and in some cases there are missing responses. To avoid introducing bias into the cohort, we used dummy variables to code missing information rather than excluding non-respondents outright. Finally, the cohort itself is limited by follow-up and some persons have as few as 4 years of follow-up (with a maximum follow-up of 15 years).

## Conclusions

We provided an update to the Canadian Community Health Survey-Mortality cohort, with a new linkage of the survey respondents to death records, inclusion of additional survey cycles, an extension of the annual residential history and mortality follow-up period, a finer scale of air pollution exposure, time-varying contextual covariates, and the inclusion of immigrants who have lived in Canada for 10–20 years (rather than only those who have been in Canada for 20+ years). The risk of non-accidental mortality from ambient PM_2.5_ was found even at low levels although the hazard ratio was attenuated in models that included other pollutants (NO_2_, O_3_, and O_X_). The PM_2.5_-mortality association displayed a supra-linear concentration-response curve. The inclusion of behavioural covariates that could confound the PM_2.5_-mortality association (fruit and vegetable consumption, leisure exercise frequency, alcohol consumption, and smoking behaviours) did not appear to impact hazard ratios. Hazard ratios were higher for males, those aged 65 or less, and non-immigrants.

## Data Availability

The datasets generated and analysed in this study are not publicly available due to privacy and confidentiality standards stated in the Statistics Act.

## Abbreviations

-2LL: (− 2) Log likelihood
AIC: Akaike information criterion
AOD: Aerosol optical depth
AQMS: Air quality management system
BMI: Body mass index
CA: Census agglomeration
CanCHEC: Canadian Census Health and Environment Cohort
Can-MARG: Canadian Marginalization Index
CCHS: Canadian Community Health Survey
CI: Confidence interval
CMA: Census Metropolitan Area
CR: Concentration-response
EPA: Environmental Protection Agency
HEI: Health Effects Institute
HR: Hazard ratio
ICD: International Classification of Diseases
NAPS: National Air Pollution Surveillance
O_X_: Oxidant capacity
PM_2.5_: Fine particle matter
ppb: Parts per billion
S.D.: Standard deviation
SBC: Schwarz Bayesian Criterion
SCHIF: Shape constrained health impact function
SDLE: Social data linkage environment
